# Bacterial DNA patterns identified using paired-end Illumina sequencing of 16S rRNA genes from whole blood samples of septic patients in the emergency room and intensive care unit

**DOI:** 10.1186/s12866-018-1211-y

**Published:** 2018-07-25

**Authors:** Monica Martins Pereira Faria, Brent Warren Winston, Michael Gordon Surette, John Maynard Conly

**Affiliations:** 1 Department of Microbiology, Immunology and Infectious Diseases, Cumming School of Medicine, University of Calgary, Calgary, AB T2N 4N1 Canada; 2 Department of Medicine, Cumming School of Medicine, University of Calgary, Calgary, AB T2N 4N1 Canada; 3 Department of Pathology and Laboratory Medicine, Cumming School of Medicine, University of Calgary, Calgary, AB T2N 4N1 Canada; 4 Department of Critical Care, Cumming School of Medicine, University of Calgary, Calgary, AB T2N 4N1 Canada; 5 Calvin, Phoebe and Joan Snyder Institute for Chronic Diseases, University of Calgary, Calgary, AB T2N 4N1 Canada; 6 O’Brien Institute for Public Health, University of Calgary, Calgary, AB T2N 4N1 Canada; 7 Farncombe Family Digestive Health Research Institute, McMaster University, Hamilton, ON L8S 4K1 Canada; 8 Department of Medicine and Biochemistry, Faculty of Health Sciences, McMaster University, Hamilton, ON L8S 4K1 Canada; 9 Department of Biomedical Sciences, Faculty of Health Science, McMaster University, Hamilton, ON L8S 4K1 Canada; 10 Foothills Medical Centre, Alberta Health Services, Room AGW5, 1403 29th Street NW, Calgary, AB T2N 2T9 Canada

**Keywords:** Sepsis, Illumina, 16 s rDNA sequencing, Molecular profiling, Bloodstream infections

## Abstract

**Background:**

Sepsis refers to clinical presentations ranging from mild body dysfunction to multiple organ failure. These clinical symptoms result from a systemic inflammatory response to pathogenic or potentially pathogenic microorganisms present systemically in the bloodstream. Current clinical diagnostics rely on culture enrichment techniques to identify bloodstream infections. However, a positive result is obtained in a minority of cases thereby limiting our knowledge of sepsis microbiology. Previously, a method of saponin treatment of human whole blood combined with a comprehensive bacterial DNA extraction protocol was developed. The results indicated that viable bacteria could be recovered down to 10 CFU/ml using this method. Paired-end Illumina sequencing of the 16S rRNA gene also indicated that the bacterial DNA extraction method enabled recovery of bacterial DNA from spiked blood. This manuscript outlines the application of this method to whole blood samples collected from patients with the clinical presentation of sepsis.

**Results:**

Blood samples from clinically septic patients were obtained with informed consent. Application of the paired-end Illumina 16S rRNA sequencing to saponin treated blood from intensive care unit (ICU) and emergency department (ED) patients indicated that bacterial DNA was present in whole blood. There were three clusters of bacterial DNA profiles which were distinguished based on the distribution of *Streptococcus, Staphylococcus*, and Gram-negative DNA. The profiles were examined alongside the patient’s clinical data and indicated molecular profiling patterns from blood samples had good concordance with the primary source of infection.

**Conclusions:**

Overall this study identified common bacterial DNA profiles in the blood of septic patients which were often associated with the patients’ primary source of infection. These results indicated molecular bacterial DNA profiling could be further developed as a tool for clinical diagnostics for bloodstream infections.

**Electronic supplementary material:**

The online version of this article (10.1186/s12866-018-1211-y) contains supplementary material, which is available to authorized users.

## Background

Sepsis refers to a systemic inflammatory response resulting from pathogenic microorganisms invading normally sterile tissues, fluids or body cavities [1]. It is often triggered by infections which have spread systemically as well as primary bloodstream infections [1]. Although any microbial agent can be implicated in sepsis, over 80% of bloodstream infections are attributed to bacteria [2–6]. The most commonly isolated bacteria from sepsis related bloodstream infections are *Staphylococcus aureus,* coagulase-negative *Staphylococci* (CoNS), *Enterococcus* species, *Escherichia coli*, and *Pseudomonas aeruginosa* [3].

Currently, sepsis bloodstream infections are primarily considered as a monomicrobial infection with rare cases of polymicrobial sepsis [7, 8]. However, these results are based on clinical diagnostic blood culture confirmed infections, which currently represents a minority of sepsis cases. We previously described a novel approach of extracting bacterial DNA from saponin-treated whole blood for use in 16S rRNA bacterial DNA analysis with Illumina sequencing [9]. Case study analysis revealed successful application of this novel approach to blood samples from septic patients in the intensive care unit (ICU). In this study, whole blood samples from expanded cohorts of ICU and emergency department (ED) patients presenting with clinical manifestations of sepsis were analyzed. The goal was to determine if molecular sequencing of bacterial DNA in the bloodstream correlated to clinical infection. Bacterial DNA profiles were analyzed alongside relevant blood culture and clinical data. This strengthened the interpretation of the DNA sequencing data as there was good concordance between the principal bacterial DNA recovered and other cultivation based data. This study supports the use of molecular profiling to augment blood culture diagnostics for identification of bacteria involved in bloodstream infections. In addition, the sensitivity of next-generation sequencing also allowed for detection of polymicrobial infections that are likely under-represented using culture-based enrichment methodology.

## Methods

### Study design

This work was conducted under the aegis of the Alberta Sepsis Network, a multi-year prospective cohort study designed to gather clinical, laboratory, and immunologic data on adult and pediatric patients admitted to the ED or the ICU with a provisional diagnosis of sepsis. Samples were collected from 2010 to 2014 at two hospitals in Calgary, Alberta, Canada. The date on which samples were collected was not provided to protect patient identity. Adult patient enrolment criteria included individuals 18 years or older admitted to the ICU of the Foothills Medical Center who met the published criteria for systemic inflammatory response syndrome (SIRS) and clinical suspicion or confirmation of infection within the first 24 h of admission or within the first 24 h of a newly acquired infection [10, 11]. SIRS criteria included; body temperature > 38 °C or < 36 °C, heart rate > 90/min, evidence of hyperventilation by respiratory rate > 20/min or PaCO2 < 32 mmHg, and white blood cell count > 12,000 cells/μl [11]. At the time of sample collection, the quick sequential organ failure assessment (qSOFA) criteria were not in clinical use [10]. The SOFA score was not regularly collected at the time of sampling, which was prior to the implementation of sepsis-3, but was available for the majority of patients admitted to the ICU [11]. As such, it was not used as an enrolment criterion. Exclusion criteria included patients in which life supportive care was deemed to be inappropriate. Adult ED patients were enrolled if they were over 18 years of age, and within the first 24 h of admission to the ED, two or more SIRS criteria and clinical suspicion or confirmation of infection. Pediatric ED patients were enrolled at the Alberta Children’s Hospital, Calgary, Alberta if they met the following criteria; under the age of 18, greater than two SIRS criteria present, clinical suspicion or confirmation of infection, and antibiotic treatment ordered for the suspected or confirmed infection and ongoing supportive care was deemed to be appropriate.

Blood was also collected from 12 healthy adults as the final control. These adults were chosen since they would represent the potential for contaminating DNA from the blood collection process including skin-associated bacteria or bacterial DNA present in the sterile vacutainers [12]. The results from these samples were previously reported [9].

### Sample collection

Sample collection for this study was done as previously described [9] using agreed upon standard operating procedures. Trained and licensed nurses or phlebotomists collected whole blood and biological samples.

Whole blood samples used for analysis were obtained on Day 1 of ICU admission for sepsis or during presentation to ED with suspected sepsis. Based on the ASN guidelines for blood collection, a maximum of 4 ml of blood and 2 ml of blood were collected from adult and paediatric patients, respectively. Blood was collected from a fresh peripheral venous vascular injection into sterile K_2_EDTA spray coated vacutainers under aseptic techniques (BD Diagnostics, Mississauga, ON). For patients admitted to the ICU, samples were collected from central arterial or venous lines which were inserted within the first 12 h of ICU admission under aseptic technique [13].

### Patient demographics, laboratory and clinical data

Clinical and diagnostic laboratory data was collected following enrolment. Data was considered relevant to the sample if collected within a 24-h period prior to or after enrolment in the study. Clinical data was obtained from the Alberta Sepsis Network database which included patient demographics, admitting diagnosis, APACHEII score [14] and the sepsis-related organ failure assessment (SOFA) score [15]. No ancestry data was collected as it is not part of patient charts in Canada. Each patient was identified only by a unique identifier based on the site in which the sample was obtained; FED samples represented adult ED patient samples, ASN samples represented adult ICU patient samples, ASNC represented adult healthy control samples, and AERG represented pediatric ED patient samples. Clinical laboratory results were collected from Calgary Laboratory Services and included all diagnostic cultures done that were relevant to the patient’s clinical presentation as well as all pharmacy related data for therapy administered.

### Saponin treatment and DNA extraction from whole blood

Blood samples noted above were then processed with a custom saponin digestion prior to DNA extraction protocol. Methods for both steps were performed as outlined in Faria et al.*,* (2015) [9]. Briefly, lysis of 1.5 mL of whole blood was achieved using 0.85% saponin (Sigma-Aldrich, USA). Lysed products were removed by centrifugation at 20,800 rcf for 15 min. Remaining cells were washed 3× with 1 ml sterile DNase/RNase free double distilled water (Life Technologies, Burlington, ON, Burlington, ON) [9]. Cells were resuspended in 500 μl sterile PBS for storage prior to DNA extraction. The extraction protocol was outlined in Faria et al.*,* (2015) and included extensive cell lysis using both lysozyme and mutanolysin (Sigma-Aldrich, Oakville, ON), RNaseA treatment (Life Technologies, Burlington, ON), proteinase K treatment (Invitrogen, Life Technologies, Burlington, ON) and DNA separation with phenol-chloroform-isoamyl (Life Technologies, Burlington, ON). Final DNA concentration and purification was done using the Zymo DNA Clean & Concentrator™-25 (Zymo Research, Irvine, CA) column containing 200 μl of ChIP DNA Binding Buffer (Zymo Research, Irvine, CA) [9].

### 16S rRNA gene bacterial community profiling with paired-end Illumina

Bacterial profiling of the v3 variable region of the 16S rRNA gene was carried out as described previously [9]. The primers used with modifications including the addition of Illumina multiplexing, bridge amplification and sequencing regions were 341F (5’CCTACGGGAGGCAGCAG3’) and 518R (5’ATTACCGCGGCTGCTGG3’) [9]. The resulting PCR products were amplified in triplicate as previously outlined [9]. Samples were sequenced using the Illumina MiSeq personal sequencer (Illumina Incorporated, USA) at the McMaster Genomics Facility (Hamilton, ON, Canada) and image analysis, base calling, and error estimation were completed using the Illumina Analysis Pipeline (version 2.6) [16]. Briefly, pooled DNA libraries were tested with the Agilent BioAnalyzer High Sensitivity DNA chip. qPCR was performed as previously described [9, 17]. The 16S rRNA gene v3 region pools were then sequenced, using previously published primers, in the forward and reverse direction on the Illumina MiSeq instrument [9, 17, 18]. Illumina’s Casava software (version 1.8.2) was used to demultiplex each run [9, 17]. Each illumina run included a no-template control sample as an internal control to ensure there was no contamination. The sequencing data was processed with custom, in-house standardized workflow and Perl scripts [9, 18]. Primer removal and trimming was carried out with Cutadapt [19] and paired-end sequences alignment and quality filtering was carried out using PANDAseq [20]. Chimera, singletons, contamination and human DNA was removed during the data filtering steps. The “noRoot” OTU was removed as it represented non-bacterial DNA amplification due to well document 16S primer cross-reactivity to human DNA [21]. Please refer to Additional file 1: Table S1 for OTUs removed during filtering. Following filtering of these sequences, a cut-off of 500 reads per sample was applied as the lowest level of abundance required for analysis.

### Taxonomic identification and diversity measures

Taxonomic summaries and subsequent analysis were done using QIIME version 1.7.0 [22]. Operational taxonomic units (OTUs) clustering and analysis of taxonomic summaries was done as previously described [9]. Briefly, operational taxonomic units (OTUs) clustering at a threshold of 97% sequence similarity was carried out using AbundantOTU+ [23]. Taxonomic identification was assigned using the Ribosomal Database Project classifier [24] using the Greengenes reference database, February 4th 2011 release [25] as a training set. QIIME computational analysis pipeline was used for community analysis [22]. Beta-diversity was used to examine variation between DNA profiles from different samples. Both weighted and unweighted UniFrac distances were used for clustering of the samples which were visualised using principal coordinate analysis (PCoA) [26, 27]. KiNG version 2.21 visualization software was used for PCoA plots [28]. Composite unweighted pair group method with arithmetic mean (UPGMA) hierarchal clustering of the sequencing data was done with weighted Unifrac distance metrics. Jackknife beta-diversity on evenly re-sampled OTU tables was applied using weighted UniFrac distance to validate the strength of UPGMA clustering [26].

The representative sequence for each OTU was also aligned to 16S rRNA sequences using the HOMD database (www.homd.org) and to the National Center for Biotechnology-Basic Local Alignment Search Tool (NCBI-BLAST, http://blast.ncbi.nlm.nih.gov/Blast.cgi) megablast nucleotide search tool.

In addition, PCR and illumina sequencing was performed on all the reagents and buffers used in the saponin blood-treatment and the DNA extraction protocol. These results were previously discussed [9].

## Results

### Patient demographics and admission results

Based on sequencing criteria discussed in the next section, not all patient blood samples processed were included in the analysis. Of the 52 ED blood samples collected, 12 were analyzed (mean age 50 years (± 13.18 SD). The predicted sources of infection were lung (4/12), genitourinary (2/12), skin soft-tissue (2/12), joint/bone (1/12), endovascular (2/12), and one unknown (Table 1). From the pediatric ED blood samples cohort, 9 of 28 samples were analyzed (mean age + 4 years (± 2.87 SD). The predicted sources of sepsis were pneumonia (3/9), intra-abdominal infection (3/9), meningitis (1/9), and two unknown (Table 1). A positive blood culture was identified in 67% of the adult ED patients and in 11% of the pediatric ED patients included in this study (Table 1). The healthy blood samples came from healthy adults and were discussed previously [9].

**Table 1:** Demographics of samples collected from adult ED (FED) patients and samples collected from pediatric ED (AERG) patients

Patient	Age Range	Gender	SIRS (1–4)	Primary Focus of Infection	Blood Culture	Top OTU (s)
Adult ED Samples						
FED31	30–40	M	2	Endovascular	Negative	*Anaerococcus*, *Staphylococcus*
FED56	70–80	M	4	Skin or soft tissue	Unknown	*Bacillaceae*
FED7	30–40	M	4	Catheter related	*Staphylococcus aureus*	*Staphylococcus*
FED36	50–60	M	4	Endovascular	*Serratia marcescens*	*Serratia*
FED14	40–50	F	3	Skin or soft tissue	*Staphylococcus aureus*	*Streptococcus*, Gammaproteobacteria
FED42	50–60	M	2	Lung	*Streptococcus pneumoniae*	*Escherichia*, Gammaproteobacteria
FED39	60–70	F	3	Unknown	Group B *Streptococcus*	*Escherichia*, *Streptococcus*
FED44	40–50	F	2	Lung	*Streptococcus pneumoniae*	*Enterobacteriaceae*, *Klebsiella*
FED15	60–70	M	2	Bone/Joint	Negative	*Streptococcus*, *Bacillus*
FED4	40–50	F	4	Urinary Tract	*Escherichia coli*	Gammaproteobacteria
FED57	40–50	F	3	Lung	Unknown	*Streptococcus, Actinomycetales*
FED34	40–50	M	3	Lung	*Streptococcus pneumoniae*	*Lactococcus*, *Streptococcus*
Pediatric ED Samples						
AERG2.106	2–3	F	2	Pneumonia	Negative	*Streptococcus, Escherichia, Staphylococcus*
AERG2.102	4–5	F	3	Appendicitis	Negative	*Staphylococcus, Streptococcus*
AERG2.113	4–5	F	3	Meningitis	Negative	*Staphylococcus, Streptococcus*
AERG1.106	2–3	F	2	Pneumonia	Negative	*Enterobacteriaceae, Streptococcus*
AERG2.043	7–8	F	2	Appendicitis	Negative	*Staphylococcus*
AERG2.076	10–11	F	2	Duplicate Cyst	Negative	*Staphylococcus*
AERG2.205	2–3	M	2	Pneumonia	Gram-positive cocci resembling *Staphylococcus*	*Bacillaceae, Staphylococcus, Moraxella, Enterococcus, Clostridium*
AERG2.235	No data	No data	No data	No data	Negative	*Bacillaceae, Staphylococcus, Moraxella, Enterococcus, Clostridium*
AERG2.198	3–4	F	4	No data	Negative	*Bacillaceae, Staphylococcus, Moraxella, Enterococcus, Clostridium*

Of the 116 ICU patient blood samples collected, 54 were used for analysis based on parameters for DNA sequencing depth outlined in a subsequent section. The clinical data upon ICU admission for the 54 patient samples used included the patients’ age, sex, APACHE II score, SOFA score, the ICU length of stay (LOS), and outcome (Table 2). Summary statistics of clinical parameters for ICU patients is available in Additional file 2: Table S2. Briefly, the mean age was 58 years (SD 15.62) with 51.9% of patients being male. The average admitting APACHE II score, a measure of disease severity [14], was 22.9 (SD 7.1). The SOFA score, a measure of organ failure [15], average was 10 (SD 4.1). With respect to mortality, 9 of 54 (17%) died during their admission. The principal sources of infection were lower respiratory tract infections *n* = 18 (33%) patients and gastrointestinal infections *n* = 16 (30%); there were *n* = 4 (7%) who had septic shock. A positive blood culture result was present in 30% of the ICU patients included in this study.

**Table 2:** Admissions data for the adult ICU patients in Groups 1–3

Sample	Age Range	Gender	Admitted From	Admitting Diagnosis	Admitting APACHE II	Max SOFA	ICU Outcome
Group 1-A							
ASN455	40–50	F	In-patient	Sepsis-Unknown	29	17	Dead
ASN350	40–50	M	Other	Bacterial pneumonia	19	18	Dead
ASN349	60–70	F	In-patient	Intracranial abscess	26	8	Alive
ASN452	70–80	M	ED	Bacterial pneumonia	13	5	Alive
ASN469	60–70	M	OR	Surgery for cellulitis	27	10	Alive
ASN470	60–70	M	In-patient	Sepsis-Gastrointestinal	34	17	Dead
ASN465	70–80	F	In-patient	Cardiac arrest, post-kidney transplant	28	12	Alive
ASN463	70–80	F	In-patient	Congestive heart failure	28	7	Alive
Group 1-B							
ASN366	50–60	M	OR-Emergency	Tonsil or pharyngeal infection	19	10	Alive
ASN357	80–90	F	Other	Septic shock	28	8	Alive
ASN376	20–30	M	OR-Emergency	Haemothorax or haemopneumothorax	20	7	Alive
ASN368	60–70	M	OR-Emergency	Leaking biliary anastamosis	11	7	Alive
ASN294	60–70	F	Other	Self poisoning with sedatives or hypnotics	10	8	Alive
Group 2- AI							
ASN167	30–40	F	ED	Hepatic abscess	15	5	Alive
ASN168	50–60	F	In-patient	Bacterial pneumonia	31	11	Alive
ASN475	70–80	F	In-patient	Gastrointestinal abscess	18	7	Alive
ASN438	50–60	F	ED	Pneumonia-Other	21	6	Alive
ASN429	70–80	F	In-patient	Respiratory cause	16	10	Alive
ASN315	20–30	M	OR-Emergency	Necrotizing fasciitis and septic shock	7	5	Alive
ASN363	30–40	M	ED	Septic shock	16	12	Alive
Group 2-AII							
ASN338	20–30	M	OR-Emergency	Traumatic rupture or laceration of liver	30	15	Alive
ASN300	60–70	F	OR-Emergency	Small bowel infarction	24	16	Alive
ASN292	60–70	M	OR-Emergency	Septic shock	34	16	Alive
ASN297	50–60	F	OR-Emergency	Oesophageal or gastro-oesophageal tumour	24	10	Alive
ASN328	20–30	M	ED	Self poisoning with narcotics	22	11	Alive
ASN473	70–80	M	ED	Bacterial pneumonia	32	11	Dead
ASN420	50–60	F	ED	Bacterial pneumonia	10	6	Alive
Group 2-AIII							
ASN379	70–80	M	In-patient	Pneumonia	28	12	Alive
ASN371	50–60	M	Other	Bleeding duodenal ulcer	21	1	Alive
ASN381	50–60	F	OR-Emergency	Necrotizing fasciitis and bacterial pneumonia	16	4	Alive
ASN432	50–60	F	ED	Bacterial pneumonia	20	8	Alive
ASN444	70–80	F	ED	Emphysema/bronchitis	24	4	Alive
ASN340	60–70	M	ED	Cutaneous cellulitis	15	12	Alive
ASN339	30–40	F	In-patient	Intracranial abscess	23	11	Alive
ASN343	50–60	M	In-patient	Inhalation pneumonitis (gastrointestinal contents)	27	8	Alive
Group 2-AIV							
ASN415	70–80	F	ED	Pneumonia-Other	30	9	Alive
Group 2-B							
ASN479	50–60	M	ED	Septic arthritis	19	11	Alive
Group 3-A							
ASN458	60–70	M	OR-Emergency	Bacterial pneumonia and cardiovascular surgery	40	20	Dead
ASN436	70–80	M	ED	Sepsis-Gastrointestinal	23	8	Alive
ASN440	60–70	F	In-patient	Congestive heart failure and emphysema/bronchitis	28	12	Alive
ASN451	70–80	F	OR-Emergency	Surgery for gastrointestinal perforation/rupture	27	14	Alive
ASN409	50–60	M	In-patient	Respiratory cause	22	10	Alive
ASN418	40–50	F	ED	Surgery for (resection) gastrointestinal vascular ischemia,	26	10	Dead
ASN454	70–80	M	ED	Upper gastrointestinal bleeding	ND^a^	4	Dead
ASN408	40–50	M	In-patient	Surgery for abdomen-trauma	15	5	Alive
ASN434	50–60	M	In-patient	Sepsis-Unknown	26	12	Alive
ASN424	60–70	M	In-patient	Surgery for (resection) gastrointestinal vascular ischemia,	14	12	Dead
ASN464	60–70	M	OR-Emergency	Surgery for gastrointestinal perforation/rupture	26	12	Alive
ASN348	70–80	F	OR-Emergency	Septic shock	27	12	Alive
ASN466	70–80	F	In-patient	Surgery for cholecystectomy/cholangitis (gallbladder removal)	31	9	Alive
ASN461	20–30	F	ED	Bacterial pneumonia	19	12	Alive
Group 3-B							
ASN476	60–70	M	In-patient	Septic arthritis	31	9	Alive
ASN474	40–50	F	In-patient	Sepsis-Pulmonary	29	19	Dead
ASN477	50–60	M	ED	Bacterial pneumonia	17	9	Alive

^a^No Data

### Bacterial DNA profiles of blood of septic ICU patients clustered into three groups

Prior to analysis, sequencing data was filtered to remove low diversity samples. After this the counts per sample were a minimum of 151, maximum of 41,7190 with a median of 1795.5 and mean count per sample of 14,725. Following the removal of the “noRoot” OTU, singletons, and known contaminant OTUs, the number of counts per sample decreased (Table 3). The OTUs removed from the analysis are available in Additional file 2: Table S2.

**Table 3:** Clusters, Clinical Microbiology, and OTU analysis of the adult ICU patient blood samples

Sample	Blood Culture	Other Culture^a^	“noRoot” OTU %	Top OTU(s)^b^	RepresentativeSequenceID^c^
Group 1^d^-A					
ASN455	*Group G Streptococcus*	*Group G Streptococcus*	98.8	175	*Streptococcus dysgalactiae/agalactiae*
ASN350	Negative	Negative	97.2	5	*Streptococcus pneumoniae/oralis/mitis*
ASN349	Negative	*Streptococcus intermedius*	99.4	5	*Streptococcus pneumoniae/oralis/mitis*
ASN452	Negative	Not Done	98.9	8	*Streptococcus intermedius/anginosus*
ASN469	*Campylobacter ureolyticus, Fusobacterium species*	*Enterococcus*	92.8	8	*Streptococcus intermedius/anginosus*
ASN470	VRE	VRE	74.8	8,5	*Streptococcus intermedius/anginosus, Streptococcus pneumoniae/oralis/mitis*
ASN465	Negative	VRE	95.4	8	*Streptococcus intermedius/anginosus*
ASN463	Negative	Not Done	97.4	8	*Streptococcus intermedius/anginosus*
Group 1-B					
ASN366	Negative	*Streptococcus anginosus, Prevotella species, CoNS*	91.9	2,5	*Staphylococcus aureus, Streptococcus pneumoniae/oralis/mitis*
ASN357	Negative	VRE	97.8	5	*Streptococcus pneumoniae/oralis/mitis*
ASN376	Not Done	Not Done	90.1	5,2	*Streptococcus pneumoniae/oralis/mitis, Staphylococcus aureus*
ASN368	Negative	*Klebsiella pneumoniae, Haemophilus parainfluenzae, Prevotella species*	94.8	5,2	*Streptococcus pneumoniae/oralis/mitis, Staphylococcus aureus*
ASN294	*Staphylococcus aureus*	*Staphylococcus aureus*	94.9	5	*Streptococcus pneumoniae/oralis/mitis*
Group 2-AI					
ASN167	Negative	SMG	94.1	32	Gammaproteobacteria
ASN168	Not Done	Fungal	96.9	32	Gammaproteobacteria
ASN475	*Pseudomonas aeruginosa*	*Pseudomonas aeruginosa*	98.0	15,32, 48	Proteobacteria*,* Gammaproteobacteria*, Pseudomonas* sp.
ASN438	Negative	*Legionella pneumophila*	98.4	3,4,379	*Enterobacter sp., Klebsiella sp., Legionella sp.*
ASN429	Negative	*Legionella pneumophila*	87.1	3	*Enterobacter sp.*
ASN315	Negative	Not Done	83.3	3	*Enterobacter sp.*
ASN363	*Staphylococcus aureus,* Gram-negative bacilli	*Staphylococcus aureus*	0.72	11	*Serratia marcescens*
Group 2-AII					
ASN338	Not Done	Not Done	96.8	2,101	*Staphylococcus aureus, Anaerococcus sp.*
ASN300	Negative	Fungal	92.5	32	Gammaproteobacteria
ASN292	Negative	Not Done	91.0	6,76,125	*Bacillus sp., Lachnospiraceae, Bacillus sp.*
ASN297	Fungal	Fungal	92.1	40,15, 125,	*Streptococcus sp.,* Proteobacteria*, Bacillus sp.*
ASN328	Negative	*Staphylococcus aureus, Streptococcus pneumoniae*	94.9	2	*Staphylococcus aureus*
ASN473	*Pseudomonas aeruginosa*	Not Done	92.5	15	Proteobacteria
ASN420	Negative	Not Done	97.5	2,59	*Staphylococcus aureus,* Proteobacteria
Group 2-AIII					
ASN379	Negative	Not Done	77.5	2	*Staphylococcus aureus*
ASN371	CoNS	Not Done	93.8	2,5,13	*Staphylococcus aureus, Streptococcus pneumoniae/oralis/mitis, Escherichia coli*
ASN381	Negative	*Legionella pneumophila*	94.0	13,3,2	*Escherichia coli, Enterobacter sp., Staphylococcus aureus*
ASN432	Negative	*Group A Streptococcus*	95.2	8,2,3	*Streptococcus intermedius/anginosus, Staphylococcus aureus, Enterobacter sp.*
ASN444	Negative	Not Done	99.2	8,3	*Streptococcus intermedius/anginosus, Enterobacter sp.*
ASN340	*Group C Streptococcus*	Not Done	99.3	2,13	*Staphylococcus aureus, Escherichia coli*
ASN339	Negative	Not Done	98.6	2,3	*Staphylococcus aureus, Enterobacter sp.*
ASN343	Negative	Fungal	99.7	2,15	*Staphylococcus aureus,* Proteobacteria
Group 2-AIV					
ASN415	Negative	Not Done	93.3	97	*Prevotella melaninogenica*
Group 2-B					
ASN479	Negative	*Finegoldia magna*	99.2	81	*Finegoldia magna*
Group 3-A					
ASN458	MRSA	MRSA	84.2	2	*Staphylococcus aureus*
ASN436	Negative	VRE	92.5	2	*Staphylococcus aureus*
ASN440	*Enterococcus faecium*	Not Done	97.2	2	*Staphylococcus aureus*
ASN451	Not Done	Not Done	99.5	2	*Staphylococcus aureus*
ASN409	Not Done	Not Done	98.6	2	*Staphylococcus aureus*
ASN418	Not Done	Not Done	87.1	2	*Staphylococcus aureus*
ASN454	Negative	Not Done	98.4	2	*Staphylococcus aureus*
ASN408	Not Done	Not Done	80.2	2	*Staphylococcus aureus*
ASN434	*Pseudomonas aeruginosa*	Not Done	8.3	2	*Staphylococcus aureus*
ASN424	*Bifidobacterium species*	Not Done	88.8	2	*Staphylococcus aureus*
ASN464	Not Done	Not Done	76.2	2,8,5	*Staphylococcus aureus, Streptococcus intermedius/anginosus, Streptococcus pneumoniae/oralis/mitis*
ASN348	Negative	*Micrococcus species, Streptococcus viridians group*	98.2	2,5	*Staphylococcus aureus, Streptococcus pneumoniae/oralis/mitis*
ASN466	Negative	*CoNS, Coryneform bacilli, Candida parapsilosis*	92.8	2,8,13	*Staphylococcus aureus, Streptococcus intermedius/anginosus, Escherichia coli*
ASN461	*Fusobacterium necrophorum*	*Fusobacterium*	90.8	2,72	*Staphylococcus aureus, Fusobacterium*
Group 3-B					
ASN476	*Escherichia coli*	Not Done	80.2	6,2,19	*Bacillus sp., Staphylococcus aureus, Lysinibacillus sp.*
ASN474	Negative	Not Done	32.6	6,2,17	*Bacillus sp., Staphylococcus aureus, Moraxella sp.*
ASN477	Negative	Not Done	96.6	6,2,32	*Bacillus sp., Staphylococcus aureus,* Gammaproteobacteria

^a^Refers to any other clinical diagnostic culture results that pertained to that patient within 24 hours of whole blood collection

^b^OTU number that represented most sequences identified and aligned in the Illumina analysis

^c^Results from alignment of the top OTU representative sequence to curated 16S rRNA databases

^d^Based on composite UPGMA trees generated using weighted UniFrac and jackknife resampling

**Fig. 1:**
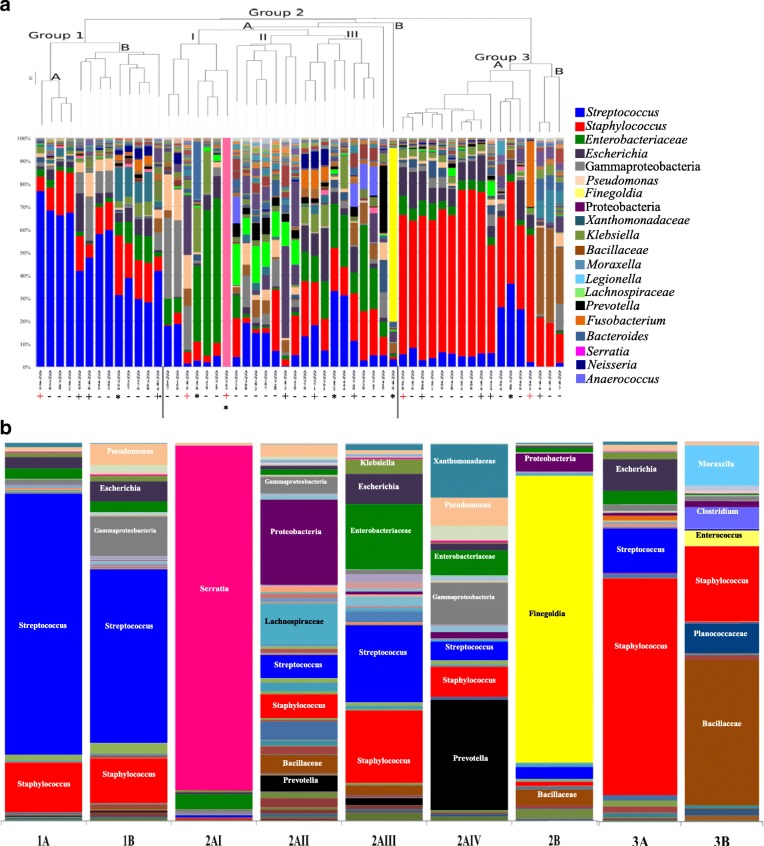
Taxonomic profiles of whole blood samples from septic ICU patient. Septic whole blood samples collected from ICU patients clustered into three groups based on their taxonomic bacterial DNA profiles. Taxonomic profiles of whole blood samples with 500 or more sequences and clustered using weighted UniFrac (54 patients). A composite unweighted pair group method with arithmetic mean (UPGMA) tree of all the samples was generated with the profiles ordered based on their placement in the UPGMA tree (**a**). Three groups of SB samples were clearly identified. Group 1 was defined by the abundance of *Streptococcus* in the profile, Group 2 by the abundance of Gram-negative OTUs, and Group 3 by the abundance of *Staphylococcus*. Blood culture results for each sample are indicated below the sample. Blood culture positive but discordant from molecular sequencing are indicated by (+), blood culture positive with concordance to sequencing by (a red +), and blood culture negative (−). Samples with a (*) are those with molecular profile results that are supported by other clinical culture data. The average taxonomic profile for the cluster groups shows the breakdown of the bacterial DNA distribution in each taxonomic cluster group (**b**)

Phylogenetic relationships in the ICU patient samples with at least 500 sequences were analyzed. Beta-diversity was assessed using jackknife analysis equally resampled OTU tables to ensure clustering was consistent [27]. Hierarchal clustering based on UniFrac was visualized as UPGMA phylogenetic trees, a well supported method for visualization of next-generation sequencing data [9, 17, 18, 22]. Due to low sequencing depth, 62 patient samples were not clustered. The taxonomic profiles of the remaining 54 patients (of the original 116) samples with a sequencing depth above 500 were clustered into three main groups (Fig. 1a). As indicated, the “noRoot” OTU was removed from analysis as it represented human DNA. When the “noRoot” OTU sequence was aligned in the NCBI-BLAST database the alignments were to mitochondrial DNA or to eukaryotic sequences. The proportion of non-bacterial “noRoot” OTU in the septic blood samples ranged from 99.98 to 0.007% with the average being 92.4% (Table 3).

For the 54 samples used in the analysis, the sequences per sample ranged from a minimum number of sequences per sample of one and a maximum of 166,596. OTUs that were detected less than 10 times in the population were excluded resulting in 460,386 sequences representing 355 OTUs. These OTUs clustered into 141 taxonomically distinct groups with the reference sequence reflecting the maximum level in which the RDP Classifier would, with confidence, identify the OTU [24].

Three clusters of DNA profiles were identified in the ICU sample cohort (Fig. 1a). Group 1 OTU profiles were distinguished by the abundance of *Streptococcus* DNA with two clades. The Group 1A samples had 65% or higher relative abundance of *Streptococcus* and the Group 1B samples with less than 65% but greater than 30% *Streptococcus* DNA (Fig. 1b). Using the representative sequence for each dominate OTU, further classification of the *Streptococcus* was predicted. Four of the patients had species of *Streptococcus* Mitis Group (*S. pneumoniae/mitis/oralis*) as the principal OTU, four had the *Streptococcus* Anginosus/Milleri *“*group*”* as the principal OTU, one had a *Streptococcus dysgalactiae/agalactiae* OTU, and an additional three had a similar abundance of a *Streptococcus* Mitis Group OTU and *Staphylococcus aureus* OTUs, and one patient had similar abundance of the *Streptococcus* Anginosus/Milleri OTU and the *Streptococcus* Mitis Group (Table 3).

Group 2 ICU patient blood samples had the greatest diversity in terms of taxonomic representation (Fig. 1a). A unifying trend for Group 2 was abundance of OTUs representing Gram-negative bacteria (Fig. 1b). Group 2 was further subdivided into two clades with Group 2A having four sub-groups I, II, III, and IV (Fig. 1b). In Group 2AI, most the DNA diversity was represented by the Gammaproteobacteria, Proteobacteria, and *Pseudomonas* taxonomic groups in the first clade whereas Group 2AII were represented by the *Enterobacteriaceae* and *Klebsiella* DNA (Table 3). Within Group 2AI, there was one blood sample in which the *Serratia* taxon represented 100% of the relative DNA abundance (Fig. 1b). There was only one *Serratia* OTU present in the SB samples and the representative sequence aligned to the *Serratia marcescens* 16S rRNA gene (Table 3). There was also one sample in Group 2AI, ASN438, in which *Legionella* DNA represented 25% of the relative DNA abundance. This was the only ICU patient where *Legionella* DNA was recovered (Fig. 1b). The Group 2AII samples had greater taxonomic diversity and the principal OTUs identified in the Group 2AII samples had sequence identities matching *Bacillus,* Gammaproteobacteria*, Lachnospiraceae, Xanthomonadaceae,* and *Staphylococcus* (Table 3). The Group 2AIII isolates had a mix of OTUs representing both Gram-positive and Gram-negative bacteria in equal proportions (Fig. 1a-b). Group 2AIV consisted of one patient blood sample in which the abundance of the *Prevotella* DNA, at 30%, separated in from the other Group 2 samples (Fig. 1b). Group 2B was also represented by a single sample where *Finegoldia* DNA represented 76% of the OTU abundance (Fig. 1b). The *Finegoldia* OTU aligned to *Finegoldia magna* (Table 3).

The third cluster of ICU blood samples grouped based on the *Staphylococcus* DNA abundance (Fig. 1a). Group 3A consisted of blood samples in which *Staphylococcus* represented 37–75% of the bacterial DNA amplified (Fig. 1a). The majority of samples had a *Staphylococcus* OTU that aligned to *S. aureus* (Table 3). The Group 3B clade was distinguished from 3A by the *Bacillaceae* and *Moraxella* DNA representing 25–41% and 5–14% of the molecular profiles (Fig. 1b). This was also the only group in which *Clostridium* and *Enterococcus* DNA were amplified to a detectable level in the taxonomic profiles (Fig. 1a).

**Fig. 2:**
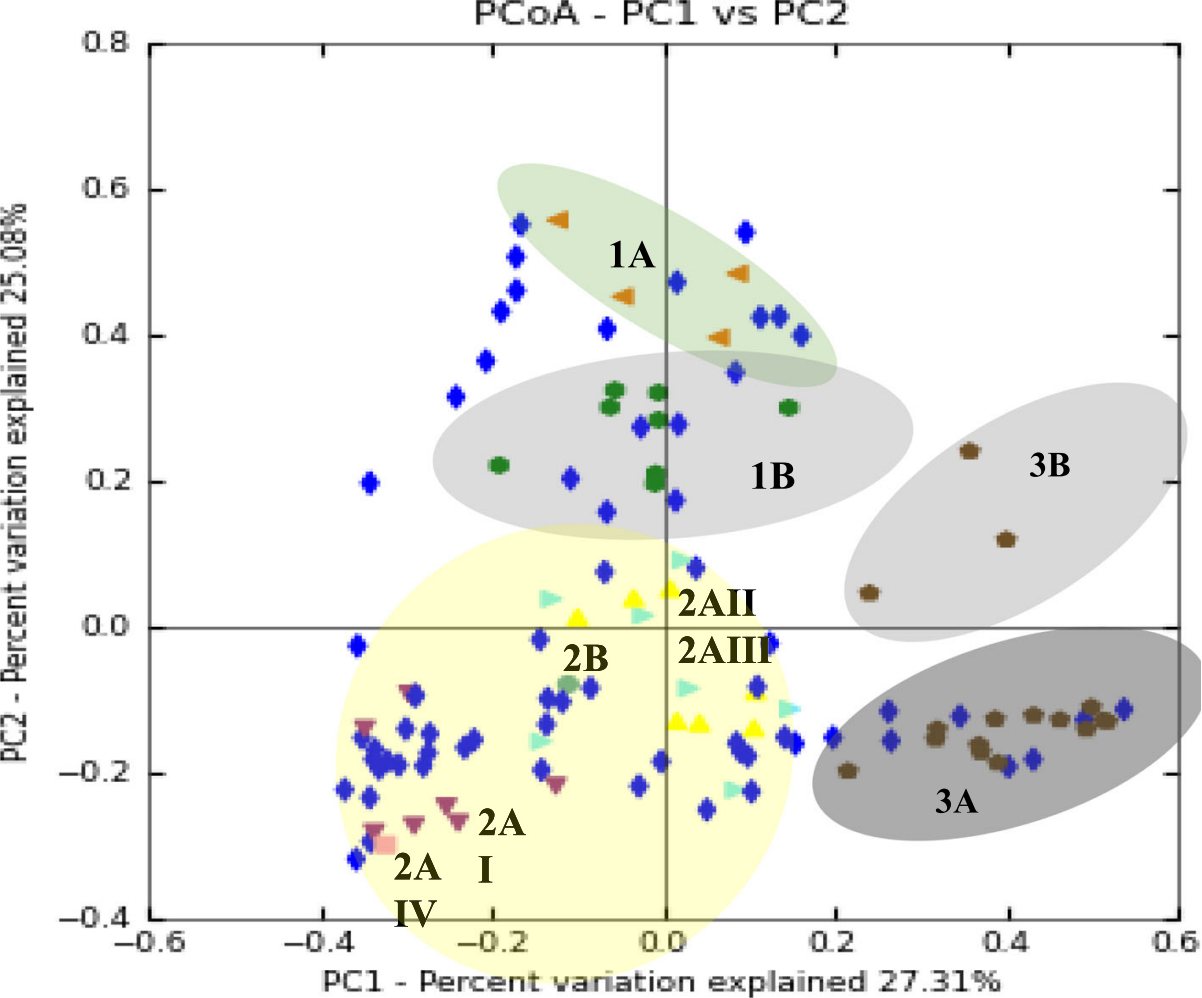
PCoA of SB samples that had low sequencing depth indicate they cluster mainly with the Group 2 samples. Principal coordinates analysis, based on weighted UniFrac was done for all blood samples from the ICU patient cohort (*n* = 116). Of these samples, 54 were used to distinguish DNA profiles into three clusters; Group 1A (orange) and Group 1B (green); Group 2AI (purple), Group 2AII (yellow), Group 2AIII (light blue), Group 2AIV (turquoise), and Group 2B (pink); and Group 3A (grey), and Group 3B (brown). The remaining 62 samples (dark blue) were overlapped with the cluster groups. Circles were added to visualize the area in the PCoA plot that each cluster group isolates occupied. The majority of the low sequence depth samples had bacterial DNA profile profiles that clustered with the Group 2 ICU blood samples and a limited number showing similarity to Group 1 (*n* = 11) or Group 3 (*n* = 8) ICU blood samples. There were 12 blood samples of low sequencing depth that did not overlap with any of the ICU blood sample clusters

Lastly, the 62 low sequence depth samples were assessed. Principal coordinates analysis (PCoA) done on low sequence depth SB samples indicated that the majority of the low sequence depth samples aligned with the three clusters of ICU patient blood samples analyzed with 12 outliers detected (Fig. 2).

### Correlation of bacterial DNA profiles to clinical microbiology data from septic ICU patients

The conventional blood culture results for the ICU patients were compared to the molecular profiles obtained in this study (Table 3). Of the 54 patients clustered, blood culture results were obtained for 46 patients with only 15 (33%) having a positive blood culture result. There was limited concordance between molecular profiling and blood culture data which was present in 5 samples (Fig. 1a). In contrast, concordance between molecular profiles and primary infection sample results was noted in several cases discussed below.

The blood sample from ASN455 had *Streptococcus* DNA representing over 75% of the bacterial DNA amplified (Table 3). The representative sequence ID for the *Streptococcus* OTU in this sample aligned to *S. agalactiae/dysgalactiae,* which are typically Group G *Streptococcus* [29]*.* This correlated with the clinical blood culture results which indicated Group G *Streptococcus* was cultivated (Table 3).

In the ASN363 sample, the *Serratia* OTU represented 100% of the relative DNA abundance (Table 3) whereas diagnostic blood culture indicated a *S. aureus* infection with Gram-negative bacilli (Table 2). Given the molecular profiling data, it could be hypothesized that the Gram-negative bacilli that failed to grow were *S. marcescens*.

ASN438 was a blood sample from a patient who was known to have a *L. pneumophila* pneumonia as part of the documented *Legionella* outbreak within the Calgary Health Region in November–December of 2012. Pleural fluid culture results for this patient were positive for *L*. *pneumophila* empyema yet blood culture was negative (Table 3). However, the molecular profiling data included the *Legionella* OTU providing evidence that *L*. *pneumophila* was likely in the bloodstream but below the threshold level to be recovered by blood culture diagnostics.

Patient ASN479 in which clinical diagnostic blood cultures were negative yet the *Finegoldia* OTU was identified in molecular profiling of their blood sample. Given the presence of *F. magna* cultured from the patient’s septic joint fluid, the molecular profiling data was suggestive of a *F. magna* bloodstream infection (Table 3).

Lastly, ASN458 patient had MRSA identified from blood culture as well as their predicted primary infection sample (Table 3). The molecular profiling of the ASN458 blood sample indicated the principal OTU had sequence alignment to *S. aureus* indicating a correlation between the molecular profiling results and the clinical culture data*.*

Overall, these cases highlighted how next-generation sequencing of DNA from septic patients could be used to detect clinically significant infections as the results correlated with the clinical data. A unifying trend was the implication of haematogenous spread of bacteria from the primary infection sources into blood even if blood cultures were negative.

### Bacterial DNA profiles from septic blood were distinct from healthy controls

**Fig. 3:**
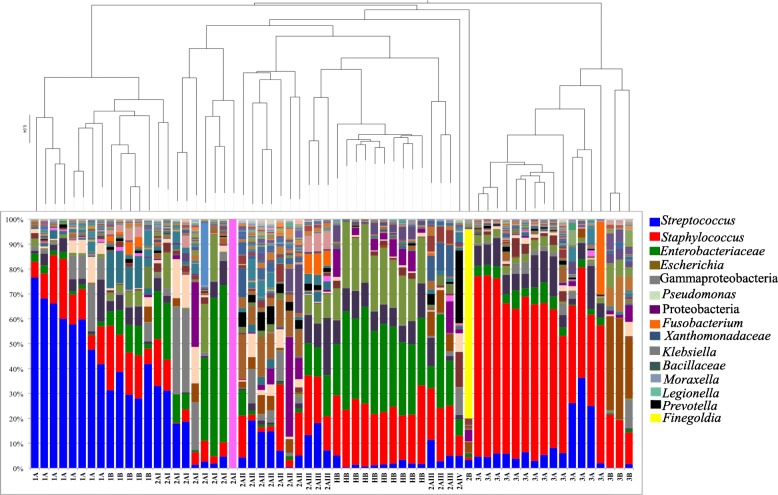
Bacterial DNA profiles from healthy adult blood samples clustered together and were phylogenetically distinct from the bacterial DNA profiles identified in blood samples from septic ICU patients. A composite unweighted pair group method with arithmetic mean (UPGMA) phylogeny of all the samples was generated with from the jackknife, weighted UniFrac beta-diversity comparison of the DNA profiles from septic ICU patient samples and healthy adult blood samples. The ICU adult blood samples were labeled based on the cluster groups identified in Fig. 1. The taxonomic cluster group’s 1A, 1B, 2AI, 2AII, 2AIV, 3A, and 3B remained intact when clustered with healthy adult blood samples. The DNA profiles from healthy adult blood samples clustered together and were a distinct clade which divided the Group 2AIII cluster. Despite this, the addition of the healthy adult blood samples did not impact the tree structure as the distribution of all three clusters of bacterial DNA profiles from septic ICU patient samples was preserved

Prior to applying this method to clinical samples, intense analysis was done to ensure bacterial DNA recovered was not a result of contamination [9]. As reported previously, DNA profiles obtained from healthy adult blood samples clustered separately from blood samples from septic ICU patients [9]. Further, the addition of the healthy blood samples to the analysis did not impact the phylogenetic tree structure distribution for cluster Group 1 or Group 3 (Fig. 3). Based on this, the bacterial DNA profiles in these groups were considered as potential bloodstream infections and not contamination. For Group 2, clusters remained intact in terms of the distribution of samples and the branching within the tree except for Group 2AIII. These samples were distinguished from the healthy control samples by the prevalence of certain OTUs including *Fusobacterium, Neisseria,* and *Anaerococcus* in the last three patients (Fig. 3). The Group 2AIII blood samples were statistically distinct from the healthy blood samples in phenetic diversity based on weighted UniFrac (PERMANOVA, *p* = 0.001) [9]. Despite this, the clustering of their molecular profiles with the control samples limited interpretation of the DNA profiles. These patients perhaps had lower bacterial DNA abundance in the sample thereby increasing the relative abundance of the contaminants in the taxonomic profile. Caution was used in the interpretation of the data such that OTU prevalence was considered significant when there was supporting clinical information.

### Common bacterial DNA patterns existed across adult and pediatric sepsis patients from the ED

**Fig. 4:**
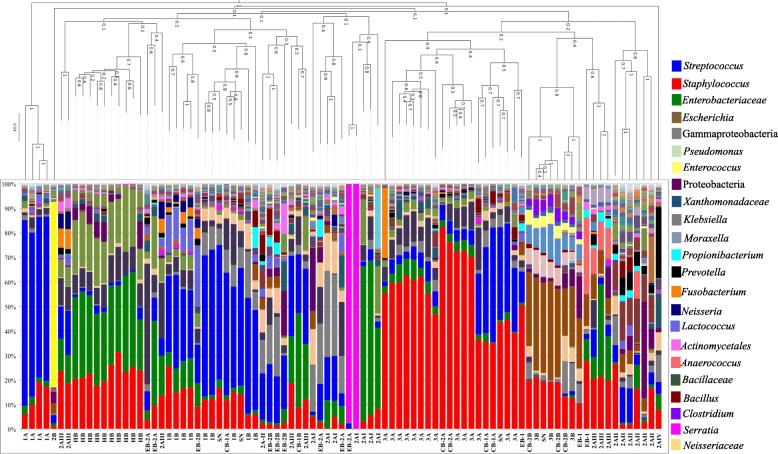
The bacterial DNA profiles of ICU and ED blood samples clustered together and separately from healthy adult blood samples. Taxonomic bacterial DNA profiles were summarized for all whole blood samples with 500 or more sequences. A composite unweighted pair group method with arithmetic mean (UPGMA) phylogeny of all the samples was generated with the profiles ordered based on their placement in the phylogenetic tree and clustered using weighted UniFrac. The samples clustered into 5 branches on the phylogenetic tree

In addition to the septic patients from the ICU, blood samples were also collected from adult and pediatric patients presenting in the Emergency Department (ED) that were suspected of sepsis. Twelve of these were analyzed further. The rationale was to determine if these ED patients had bacterial DNA profiles similar those patients admitted to ICU with clinically confirmed sepsis. The bacterial profiles from the ED patients cluster with the ICU samples into the groups described in Fig. 1 and distinct from the healthy controls (Fig. 4). As seen with the ICU patient cohort, the clusters were defined by abundance of *Streptococcus* OTUs, Gram-negative OTUs, or *Staphylococcus* OTUs. Nine ED sample profiles clustered with the Gram-negative dominant samples whereas two ED samples grouped with the *Staphylococcus* and one ED sample grouped with the *Streptococcus* dominant samples, respectively (Fig. 4).

## Discussion

The use of Illumina sequencing technology combined with a novel DNA recovery method enabled the characterization of bacterial DNA isolated from 3 to 5 ml blood samples collected from several cohorts of septic patients. Among these cohorts, the samples from patients admitted to ICU with sepsis had the highest number of samples available to examine trends. Analysis of the bacterial DNA profiles, presented as a proportion of total bacterial DNA, indicated that three common distributions were present in these samples. Association with the infection source, based on the admission diagnosis, showed the strongest correlation to the bacterial DNA profiles. The Group 1 bacterial DNA profile had OTUs representative of commensal microbiota from the upper respiratory tract or the skin in addition to *Streptococcus* as the predicted pathogen (Fig. 1, Table 2). Many of the patients in Group 1 were admitted with pneumonia, upper respiratory tract infections, abscess and cellulitis. *Streptococcus* species are recognized principal pathogens in these clinical presentations [29–35]. The Group 2 patients had diverse clinical presentations and bacterial DNA profiles representing Gram-negative organisms (Table 2, Table 3, Fig. 1b). Patients admitted with gastrointestinal infections or trauma likely developed sepsis from gastrointestinal microbiota including known Gram-negative opportunistic pathogens [36]. The remaining patients within Group 2 with abscesses or airway infections had bacterial DNA OTUs that correlated to upper airway and skin associated microbiota [31, 33, 37–41]. The Group 3 bacterial DNA profiles were distinguished by the large proportion of *Staphylococcus* OTUs (Fig. 1). These samples were obtained from patients admitted for emergency surgical interventions, joint infections, and pneumonia (Table 2). Again, the role for *Staphylococcus* as a clinical pathogen in such presentations of sepsis is well documented [31, 37, 39, 42–50]. Taken together, these data support the interpretation of these bacterial DNA profiles as representation of bacterial bloodstream infections with DNA from known pathogenic organisms recovered that correlated to the patient’s clinical presentation at the time of enrollment in the study.

The molecular profiling results provided more evidence of sepsis bloodstream infection when compared to the conventional diagnostic blood culture. For the adult ICU blood samples included in the analysis, only 33% had a positive clinical blood culture (Table 2). In comparison, bacterial DNA was recovered from all these blood samples and the bacterial DNA profiles in these samples were distinct from those recovered from the blood of healthy adult control samples (Fig. 3). While the presence of bacterial DNA in these blood samples did not indicate the presence of viable organisms, it suggested that the clinical blood cultures were under-representing the presence of bloodstream infections in this cohort. These results are comparable to similar studies using molecular diagnostic platforms have also reported under representation of bloodstream infections when blood culture diagnostics were compared to PCR based methods [13, 51–55]. This study also outlined several cases where the bacterial DNA amplified from the blood sample had good concordance with bacterial pathogens that were recovered from pertinent clinical diagnostic cultures. Overall, combining our molecular profiling analysis of the bacterial DNA patterns with a chart review of the patient clinical data including culture-based diagnostic results strengthened our interpretations of the molecular profiling results and further demonstrated the potential for this molecular-based approach to augment culture-based microbial diagnostic results.

This study also demonstrated that the bacterial DNA patterns were conserved across various subsets of septic patients. Indeed, there was similar clustering of all the clinical blood samples regardless of the patient’s presentation to ICU or ED and across both adult and pediatric cohorts (Fig. 3). In addition, this analysis confirmed two principal bacterial DNA patterns seen in the septic ICU cohort; one in which *Streptococcus* DNA was the most prevalent and one in which *Staphylococcus* DNA was the most prevalent (Fig. 3). Further analysis of the OTU distribution of *Streptococcus* indicated that the principal predicted *Streptococcus* species found in whole blood were the *Streptococcus* Anginosus/Milleri Group and *Streptococcus* Mitis Group *(S. pneumoniae/mitis/oralis)* at 33.5 and 10.59% respectively (Additional file 3: Table S3). The prevalence of the Anginosus/Milleri Group superseding that of Mitis Group was not expected given many studies suggesting *S. pneumoniae* as a principal pathogen recovered in clinical diagnostic blood culture positive bloodstream infections [56]. A recent study indicated 89% of culture-positive bloodstream infections were a result of *S. pneumoniae* [57] whereas the Anginosus/Milleri Group represented a smaller proportion of the *Streptococcus* bloodstream infections [49]. As such, these results suggested greater diversity of *Streptococcus* species in sepsis bloodstream infections than previously considered based on blood culture diagnostics. Interestingly, other studies using targeted culturing and culture-independent approaches have also demonstrated a role for the Anginosus/Milleri Group in human infections [58–61]. This study now adds new data to suggest a greater role for this group in acute bloodstream infections than previously reported [8, 49, 57].

For *Staphylococci,* the OTU with sequence alignment to *S. aureus* represented 97% of the *Staphylococcus* OTUs present in the septic ICU population (Additional file 3: Table S3). Most reports from clinical diagnostic blood culture confirmed bloodstream infections indicate *S. aureus* as the second most commonly isolated organism [56]. *S. aureus* predominate blood samples were obtained from patients with documented surgical infections (11/17), respiratory infections (5/17), and septic arthritis (1/17). Post-operative *Staphylococcus* infections have been documented in other literature reports [62, 63] and *S. aureus* is a common pathogen in respiratory infections and septic joint infections [50]. The remaining 3% of *Staphylococcus* OTUs aligned with CoNS (Additional file 3: Table S3). Most population-based assessments cluster the CoNS bloodstream infections together since clinical laboratories don’t distinguish these organisms beyond this level [49, 64]. Taken together, the molecular profiling data suggested that there might be a larger role for diverse CoNS in sepsis than is currently appreciated using clinical diagnostic blood culture approaches.

Overall, this study demonstrated the potential strengths of the molecular profiling data when evaluated alongside the patient’s admissions data and, to some extent, their culture data. The results indicated *Streptococcus* and *Staphylococcus* as principal pathogens in sepsis bloodstream. However, the prevalence of polymicrobial DNA in whole blood from septic patients suggested there could be greater propensity for polymicrobial infections in sepsis than currently appreciated using cultivation-dependent and broth-enrichment based approaches. Similar results from direct blood analysis have shown utility of molecular profiling for identification of microbial DNA and its utility as an additional tool for sepsis diagnostics [13, 54, 55].

We recognize that our study had limitations. Not all the blood samples analyzed had a sequencing depth that allowed for good interpretation of β-diversity [65]. In the blood samples the amount of bacterial DNA template was low as compared to the host template resulting in the high relative abundance of the “noRoot” OTU. This “noRoot” OTU was attributed to the well-documented erroneous amplification of human DNA in clinical samples with universal 16S rRNA gene primers. This issue has been reported since the early days of PCR [66] and is still problematic in contemporary 16S rRNA gene studies [66–68]. In this study, the abundance of “noRoot” DNA often represented a large portion of the amplified sequences in whole blood. This was unique to our study and likely reflected the low ratio of bacterial to host DNA in these samples. It is difficult to know the exact concentration of bacteria in bloodstream infections since the blood culture results only indicate the CFU/ml of bacteria after a broth-enrichment. However, in the limited number of samples where culture from saponin treated whole blood was successful, the CFU/ml were between 1 to 30 (data not shown) suggesting the concentration of bacteria would be low in the clinical samples. Following the removal of the “noRoot” reads, the samples often had a low number of remaining sequences. A reasonable cut-off was needed to ensure that differences in the taxonomic structure of samples could be identified. The strength of UniFrac beta-diversity to identify meaningful patterns in various datasets has been well documented [27]. Even in small sample size simulations (50 sequences) the UniFrac values could be used to discriminate between samples [27]. However, when the expected similarity in microbial communities among different samples was anticipated to be high, more sequencing reads were required to identify relationships [27]. It is also known that between 500 and 1000 reads/sample is sufficient, but not ideal, to distinguish differences in phylogenetic composition between two samples using beta-diversity. As such, a depth of 500 reads was selected as it permitted evaluation of more of the samples with the knowledge that the interpretation of the profiles required caution in the absence of good clinical data. When compared to other molecular profiling studies of blood our abundance threshold was significantly lower [13]. Given the difference in DNA extraction protocols, PCR amplification, sequencing platforms and analysis methods it is difficult to compare the quality of sequencing data based on abundance per sample. Prior to extensive filtering the read per sample averaged at over 14,000 reads which is in line with other molecular profiling studies [13]. The lower abundance per sample was interpreted to reflect the low ratio of bacterial DNA sequences compared to the “noRoot” human DNA (Table 2). Since this was a ratio-based issue, the use of larger blood volumes was not predicted to circumvent these limitations. Nevertheless, the removal of these DNA sequences from the taxonomic profile enabled the analysis of the remaining, low proportion, bacterial DNA in the samples. Although this resulted in many samples not being fully analyzed, PCoA analysis indicated that low sequence depth samples still clustered alongside SB samples (Fig. 2). This would suggest that most whole blood samples had similar molecular profiles to the SB samples in Fig. 1 despite lower sequencing depth.

Another limitation was that the bacterial DNA profiles reflected relative not total DNA abundance. This meant that no conclusions the quantity of bacterial DNA in these samples. Attempts to quantitate the bacterial load in the HB and SB samples, using RT-PCR, were unsuccessful due to the cross-reactivity of the 16S primers to human DNA in these samples (data not shown).

As such, the bacterial DNA profiles could indicate the taxonomic diversity in each sample but not the bacterial load. Finally, we reiterate that the molecular analysis identifies the presence of bacterial DNA not viable organisms. The isolation protocol described previously [9] should reduce the level of free DNA in the preparation and therefore these profiles should be enriched in DNA from intact cells.

Based on these limitations, it was essential that each sample was evaluated within the clinical context. Many reviews of molecular profiling strategies have highlighted the importance of analyzing molecular data in conjunction with other clinical measures of severity (i.e., APACHE, SOFA scores), markers of infection (i.e., procalcitonin), and markers of inflammation (i.e., IL-6, IL-10) [69–72]. However, our findings reveal that when the bacterial DNA patterns were aligned with the clinical data it was apparent that meaningful patterns were observed in the data. Despite this, our data also highlighted discordance between blood culture enrichment and molecular sequencing can occur. When results were discordant, there was often other clinical data to support the molecular sequencing (Fig. 1a, Table 3). In other cases, the discordance was thought to result from difference in the time of collection as well as reflecting discrepancy between a broth enrichment method to that of direct sampling [69, 73–75]. While our initial blood samples were obtained within 24 h of ICU admission or in the ED once sepsis was suspected, it did not guarantee that our samples were collected prior to initiation of antimicrobial therapy. With early antibiotic therapy being a hallmark of sepsis management as outlined in sepsis guidelines, it was predicted that the majority of our ICU patient samples were obtained after antimicrobial therapy was started whereas blood culture results are often obtained prior to antimicrobial therapy [11, 76, 77]. As such, effective antimicrobial therapy was also considered when discordance was present.

## Conclusions

The overall evaluation of a whole blood molecular profiling approach to evaluating septic bloodstream infections provided several novel findings. Overall, the bacterial DNA profiling of whole blood samples from adult and pediatric patients was correlated to a predicted bloodstream infection with either a viable organism or bacterial products in 75% of the samples analyzed in this study. In addition, the molecular profiling data predicted a greater role for polymicrobial infections in the pathogenesis of sepsis.

## Additional files

Additional file 1: **Table S1.** OTUs identified as contamination and removed from OTU table. Outlines OTUs that were identified in the data as representing contamination and removed using custom perl scripts prior to final analysis of OTU distribution in each sample. (DOCX 12 kb)

Additional file 2: **Table S2.** ICU patient demographics and clinical markers of sepsis severity. This table includes summary statistics of patient data for samples from adult ICU patients. The data was separated by the beta-diversity groups identified in the meta-analysis. Data includes gender, age, illness severity scores, length of stay and outcomes. (DOCX 13 kb)

Additional file 3: **Table S3.** *Streptococcus* and *Staphylococcus* species predicted to be present in ICU patient blood samples. This table highlights the results of the DNA alignments of the 250 bp representative sequence from the *Streptococcus* and *Staphylococcus* OTUs identified in clinical blood samples to curated 16S rRNA sequence databases. (DOCX 13 kb)

## Abbreviations

APACHE: Acute Physiology and Chronic Health Evaluation
ASN: Alberta Sepsis Network
CFU: Colony forming unit
CoNS: Coagulase-negative *Staphylococci*
ED: Emergency department
ICU: Intensive care unit
MRSA: Methicillin-resistant *Staphylococcus aureus*
OTU: Operational taxonomic unit
PCoA: Principal coordinates analysis
PCR: Polymerase chain reaction
SIRS: Systemic inflammatory response syndrome
SOFA: Sepsis related organ failure assessment
UPGMA: Unweighted pair group method with arithmetic mean
