# Supplementary material for: Bacterial DNA patterns identified using paired-end Illumina sequencing of 16S rRNA genes from whole blood samples of septic patients in the emergency room and intensive care unit
Source: BMC Microbiol. 2018 Jul 25;18:79. doi: 10.1186/s12866-018-1211-y (PMC6060528; doi:10.1186/s12866-018-1211-y)
Supplement: Supplementary file 1 — Table S1. OTUs identified as contamination and removed from OTU table. Outlines OTUs that were identified in the data as representing contamination and removed using custom perl scripts prior to final analysis of OTU distribution in each sample. (DOCX 12 kb) [file 12866_2018_1211_MOESM1_ESM.docx]

Additional file 1: Table S1. OTUs identified as contamination and removed from OTU table.

| **Taxonomic Level** | **OTU Taxonomic Identification** |
| --- | --- |
| noRoot | Eukaryotic DNA |
| Genus | *Burkholderia* |
| Genus | *Bradyrhizobium* |
| Genus | *Cupravidus* |
| Genus | *Variovorax* |
| Genus | *Aquabacterium* |
| Genus | *Sphingobium* |
| Genus | *Rhodobacter* |
| Genus | *Sphingomonas* |
| Genus | *Rhodanobacter* |
| Genus | *Thermus* |
| Genus | *Meiothermus* |
| Genus | *Janthinobacterium* |
| Genus | *Methylobacterium* |
| Genus | *Mesorhizobium* |
| Genus | *Acidocella* |
| Genus | *Chryseobacterium* |
| Genus | *Acidovorax* |
| Genus | *Delftia* |
| Genus | *Novosphingobium* |
| Genus | *Akkermansia* |
| Genus | *Paucibacter* |
| Order | *Rhizobiales* |
| Order | *Rhodobacterales* |
| Order | *Rhodospirillales* |
| Order | *Sphingomonadales* |
| Order | *Burkholderiales* |
| Order | *Oceanospirillales* |
