# Supplementary material for: Bacterial DNA patterns identified using paired-end Illumina sequencing of 16S rRNA genes from whole blood samples of septic patients in the emergency room and intensive care unit
Source: BMC Microbiol. 2018 Jul 25;18:79. doi: 10.1186/s12866-018-1211-y (PMC6060528; doi:10.1186/s12866-018-1211-y)
Supplement: Supplementary file 2 — Table S2. ICU patient demographics and clinical markers of sepsis severity. This table includes summary statistics of patient data for samples from adult ICU patients. The data was separated by the beta-diversity groups identified in the meta-analysis. Data includes gender, age, illness severity scores, length of stay and outcomes. (DOCX 13 kb) [file 12866_2018_1211_MOESM2_ESM.docx]

Additional file 2: Table S2. ICU patient demographics and clinical markers of sepsis severity.

|  | **Group 1**  **N=13** | **Group 2**  **N=24** | **Group 3**  **N=13** |
| --- | --- | --- | --- |
| **Sex** | | | |
| Female | 46.2% | 54.2% | 38.5% |
| Male | 53.8% | 45.8% | 61.5% |
| **Age (years)** | | | |
| Median | 63 | 57 | 63 |
| IQR | (53-70) | (52.75-69.5) | (53-68) |
| **APACHE II** | | | |
| Median | 26 | 21.5 | 26 |
| IQR | (19-28) | (16-28) | (22-29) |
| **SOFA-Day 1** | | | |
| Median | 7 | 7 | 10 |
| IQR | (7-10) | (4-10) | (9-12) |
| **SOFA-Max** | | | |
| Median | 8 | 10 | 12 |
| IQR | (7-12) | (6.5-12) | (9-12) |
| **ICU LOS (days)** | | | |
| Median | 5 | 6 | 6 |
| IQR | (4-7) | (4-10.25) | (4-16) |
| **ICU Outcome** | | | |
| Alive | 76.9% | 95.8% | 84.6% |
| Dead | 23.1% | 4.2% | 15.4% |
