# Supplementary material for: Bacterial DNA patterns identified using paired-end Illumina sequencing of 16S rRNA genes from whole blood samples of septic patients in the emergency room and intensive care unit
Source: BMC Microbiol. 2018 Jul 25;18:79. doi: 10.1186/s12866-018-1211-y (PMC6060528; doi:10.1186/s12866-018-1211-y)
Supplement: Supplementary file 3 — Table S3. Streptococcus and Staphylococcus species predicted to be present in ICU patient blood samples. This table highlights the results of the DNA alignments of the 250 bp representative sequence from the Streptococcus and Staphylococcus OTUs identified in clinical blood samples to curated 16S rRNA sequence databases. (DOCX 13 kb) [file 12866_2018_1211_MOESM3_ESM.docx]

Additional file 3: Table S3. *Streptococcus* and *Staphylococcus* species predicted to be present in ICU patient blood samples

| **OTU ID** | **OTU #** | **Abundance** | **Representative Sequence ID^1^** | **Match Percentage^2^** |
| --- | --- | --- | --- | --- |
| *Streptococcus* | 8 | 33.5% | *Streptococcus intermedius/anginosus* | 100 |
| *Streptococcus* | 5 | 10.6% | *Streptococcus pneumoniae/oralis/mitis* | 100 |
| *Streptococcus* | 40 | 2.3% | *Streptococcus salivarius/vestibularis* | 100 |
| *Streptococcus* | 175 | 1.6% | *Streptococcus dysgalactiae/agalactiae* | 99 |
| *Streptococcus* | 127 | 0.5% | *Streptococcus sinensis* | 100 |
| *Streptococcus* | 25 | 0.3% | *Streptococcus pyogenes* | 100 |
| *Streptococcus* | 302 | 0.06% | *Streptococcus pluranimalium* | 99 |
| *Streptococcus* | 700 | 0.06% | *Streptococcus uberis/porcinus* | 99 |
| *Staphylococcus* | 2 | 96.6% | *Staphylococcus aureus* | 100 |
| *Staphylococcus* | 44 | 2.5% | *Staphylococcus sciuri* | 99 |
| *Staphylococcus* | 82 | 0.5% | *Staphylococcus intermedius* | 99 |
| *Staphylococcus* | 144 | 0.2% | *Staphylococcus saprophyticus* | 99 |
| *Staphylococcus* | 304 | 0.1% | *Staphylococcus epidermidis* | 100 |
| *Staphylococcus* | 119 | 0.03% | *Staphylococcus aureus* | 97 |
| *Staphylococcus* | 434 | 0.02% | *Staphylococcus pseudointermedius/chromogenes* | 100 |
| *Staphylococcus* | 876 | 0.01% | *Staphylococcus aureus* | 97 |

^1^ Results from alignment of the top OTU representative sequence to curated 16S rRNA databases

^2^ Alignment between representative sequence for the OTU and the top 16S rRNA match
